# Conservation genomics of wolves: The global impact of RK Wayne’s research

**DOI:** 10.1093/jhered/esae007

**Published:** 2024-02-21

**Authors:** Marco Musiani, Ettore Randi

**Affiliations:** Dipartimento di Scienze Biologiche, Geologiche e Ambientali (BiGeA), University of Bologna, Bologna, Italy; Department of Chemistry and Bioscience, Aalborg University, Aalborg Øst, Denmark

**Keywords:** *Canis lupus*, ecological specialization, ecotypes, genetics, genomics, wolf-dog hybrids

## Abstract

RK Wayne has arguably been the most influential geneticist of canids, famously promoting the conservation of wolves in his homeland, the United States. His influence has been felt in other countries and regions outside the contiguous United States, where he inspired others, also including former graduate students and research fellows of his, to use modern molecular techniques to examine the evolutionary biology of canids to inform the conservation and management of wolves. In this review, we focus on the implications of Wayne’s work on wolves outside the United States. He envisioned a clear future for wolf conservation research, involving the study of wolves’ ecological and genetic diversity, and the description of ecotypes requiring conservation. He also documented widespread hybridization among canids and introgression of DNA from domestic dogs to wolves, a process that started dozens of thousands of years ago. His work therefore calls for innovative studies, such as examining the potential fitness benefits of introgression. Inspired by his results, for example, on the purging of deleterious alleles in small populations, wolf researchers should use novel molecular tools to challenge other conservation genetics paradigms. Overall, RK Wayne’s work constitutes a call for answers, which as scientists or citizens concerned with conservation matters, we are obliged to address, as we contribute to monitoring and maintaining biodiversity during our period of dramatic transformations of the biosphere.

## Graphical Abstract

**Graphical Abstract:**
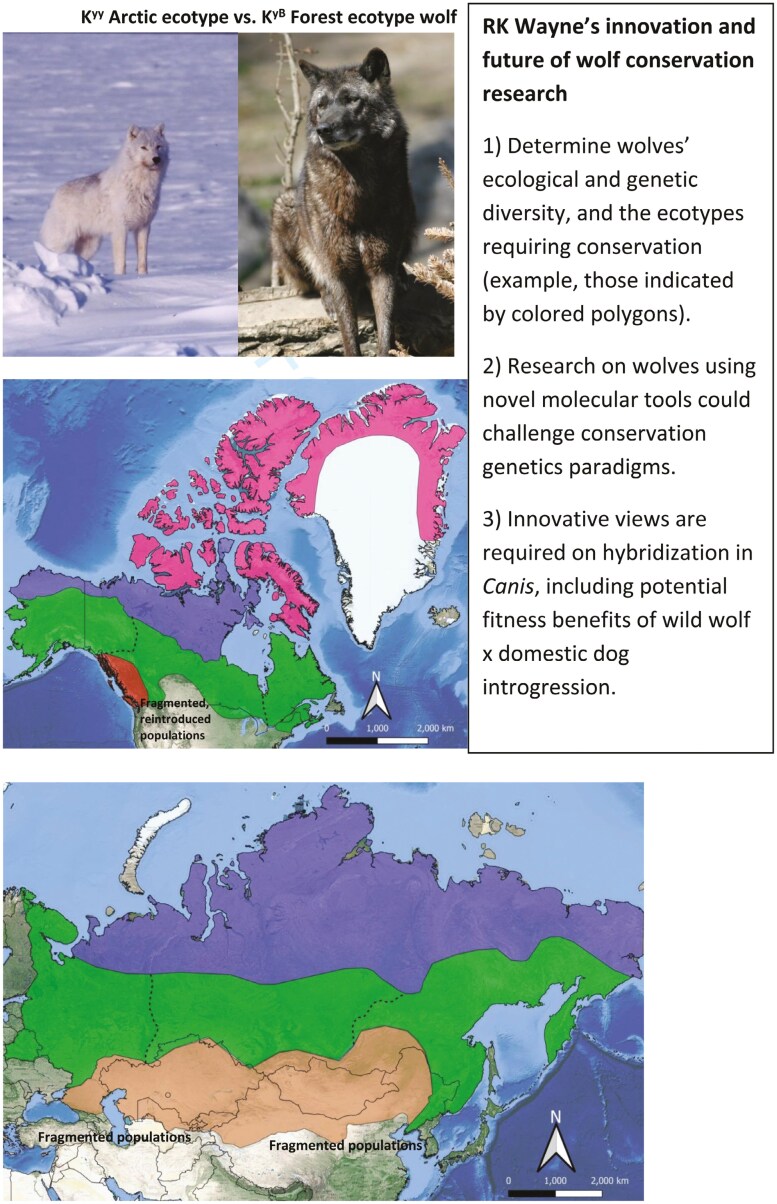

## Introduction

Robert K. Wayne has been perhaps the first and potentially the most influential conservation geneticist of gray wolves (*Canis lupus*). He produced papers on genetics of wolves throughout their range, which covers most of the Northern Hemisphere. His homeland was the contiguous United States (i.e. the “conterminous” 48 adjoining states and the District of Columbia), where he was directly engaged in conservation. In other countries and regions, researchers were truly inspired by his ground-breaking work. This international group of wolf-focused conservation geneticists included former graduate students, postdoctoral fellows, and colleagues of RK Wayne, who collaborated with him throughout his career. Overall, RK Wayne’s work inspired the use of modern genetics to examine the evolutionary biology of canids and to influence conservation and management of wolves both in the contiguous United States and other countries and regions across the world.

RK Wayne’s efforts contributed considerably to a universal understanding of the worldwide evolution of *C. lupus* and canids in general. Genetics and genomics of contemporary wolf populations were the main focus of his group’s research, while he also conducted paleogenomic studies to determine the origin of such populations. His lab advanced the use of a variety of molecular markers to study phenotypic and ecological traits with the goal of contributing to both foundational evolutionary biology and canid conservation. Graduate students have benefited from RK Wayne’s expertise, while also helping to publish scientific reports with him that will go down in the history of conservation sciences.

RK Wayne’s work on wolves in the contiguous United States, the transboundary (Canada–United States) Great Lakes region, and Eastern Canada has received immense attention, due to its often political conservation implications (Wayne and Jenks 1991; Leonard and Wayne 2008; vonHoldt et al. 2016). Therefore, in this review, we would like to pay tribute to the legacy that he has left us with, by focusing on his equally marvelous findings regarding the evolution and conservation of Eurasian and other North American wolves. His empirical research has never been separated from theoretical insights with much broader applications. Overall, RK Wayne’s work constitutes a call for answers, which as scientists or citizens concerned with conservation matters, we are obliged to address, as we contribute to monitoring and maintaining biodiversity during the current period of dramatic transformations of the biosphere.

## Paleogenomics and the emergence of contemporary wolf populations

The presence of wolves in the Northern Hemisphere has been documented throughout the last 800,000 years (Diedrich 2022). RK Wayne and coworkers (Leonard et al. 2007) used morphological, isotopic, and genetic analyses (mitochondrial DNA, mtDNA sequencing) to characterize Late Pleistocene gray wolves from West Beringia (modern-day Alaska), which was at the time connected to Eurasia by a land bridge. Such research highlighted a fossil gap, suggesting a dramatic decline in wolf populations in Beringia before the end of the Last Glacial Maximum (LGM; *c.* 20,000 YBP). Interestingly, the mtDNA haplotypes of ancient wolves are absent in contemporary wolves. In addition, ancient Beringian wolves exhibited distinct craniodental morphology, suggesting their specialization for large-sized prey, including species that then became extinct. Therefore, the disappearance of these prey species likely led to the demise of ancient Pleistocene wolves. Then, Holarctic wolf populations emerged, colonizing Eurasia and subsequently the rest of North America following the end of the LGM. This study (Leonard et al. 2007) was seminal and fostered further research on the origin and diffusion of wolf populations in North America and Eurasia.

Whole-genome analyses from RK Wayne’s group (Freedman et al. 2014; Fan et al. 2016) confirmed that extant wolf populations likely originated from a rapid expansion of wolves from Beringia. These results were corroborated by Loog et al.’s (2020) work, which analyzed mitochondrial genomes and modeled the demography of wolves over the last 50,000 years, Finally, a synthesis by Schweizer et al. (2020) confirmed RK Wayne’s vision of this rapid wolf expansion. Recent paleontological findings also provided further evidence that post-Pleistocene wolves quickly colonized the Northern Hemisphere and replaced older populations in both North America and Eurasia (Ramos-Madrigal et al. 2021).

As a result, extant mtDNA haplotypes are now widely distributed across the Northern Hemisphere (Koblmüller et al. 2016), suggesting that wolves historically dispersed through long-range movements, similar to extant wolves. However, despite this capability for long-range dispersal, extant populations exhibit significant isolation-by-distance and structuring. Thus, ecological constraints and/or geographic barriers can limit interpopulation gene flow rates, resulting in local differentiation of wolf populations (vonHoldt et al. 2011). More recently, centuries of persecution of wolves by people, which lasted until the 1970s, had led to dramatic population declines around the world (also with the genetic effects determined by RK Wayne’s studies), including the extirpation of several wolf populations (Mech 1970).

## Innovative themes in wolf conservation research

At present, wolves, similar to other terrestrial apex predators, are recovering in various regions of the world, particularly in western countries (Twining et al. 2021). However, expanding wolf populations may exacerbate conflicts with humans in densely populated regions, especially areas with livestock production (Chapron et al. 2014). Coexistence of humans and livestock with large predators, like wolves, requires creative and innovative solutions. Threats to wolf and other large terrestrial predators have been a long-lasting concern that always stimulated RK Wayne’s research. From his often premonitory studies, highly relevant suggestions have emerged, aiding to better focus conservation research and conservation planning. We would like to highlight the following three innovative themes in wolf conservation research, fully appreciating that his initial work in these directions has and will serve as a role model for work conducted by other researchers internationally.

### Determining wolves’ ecological and genetic diversity, and the ecotypes requiring conservation

RK Wayne’s methodological approach clearly promotes that wolf conservation at the continental scale must be based on an understanding of spatially meaningful genetic and adaptive variation (see Wayne et al. 1995; Wayne and Vila 2003, Kohn et al. 2006). Several concepts may be used to define such variation in the context of wildlife management, including populations, subspecies, ecotypes, conservation units, management units, and evolutionary significant units (ESUs) (see Crandall et al. 2000). Wayne and his colleagues (Crandall et al. 2000) suggested that concordant distributions of multiple, independent traits including behavioral, ecological and genetic variation of adaptive significance provide the basis for phylogenetic distinction. RK Wayne’s results on North American wolves (Schweizer et al. 2016b) and initial findings by others on Asian wolves (Werhahn et al. 2022) demonstrate that the species is not panmictic and that spatial variation in prey specialization influences spatial patterns of gene flow (Musiani et al. 2007). The apparent paradox of differentiation in such a highly mobile wide-ranging species is therefore explained by understanding the trophic ecology and social behavior of wolves.

Specialization on prey likely occurred relatively recently, starting in the Holocene, and has contributed to the ecological and genetic divergence of wolves observed today (Musiani et al. 2007; Werhahn et al. 2022). Similar to other large mammals with an ecological scope to occupy different ecological niches (Stronen et al. 2022b), wolf adaptation to different prey has shaped divergent morphologies, behaviors, and ecologies, and is reflected at the genetic level. Wolves that are morphologically, behaviorally, ecologically, and genetically different should be considered as separate ESUs to be maintained in conservation planning (Crandall et al. 2000). Therefore, RK Wayne’s results on wolf ESUs may be also applicable in other ecosystems or regions, including Asian, where wolves select differing prey species. Similar studies on wolf evolutionary units should be conducted in those regions to inform management decisions regarding wolves, for example, to make sure that wolf control does not eliminate whole ESUs. Moreover, his findings suggest that wolf management should always consider the evolutionary significance of trophic ecology (prey specialization).

This review’s analysis of how genetic work is incorporated in wolf management in North America and Asia emphasizes that regardless of government regulations or biological considerations, human attitudes play a pivotal role in the persistence of wolf populations. For example, in the former Union of Soviet Socialist Republics, USSR the task of regulating wolf populations was recognized as highly important on a national scale to reduce the damage from wolf predation on agriculture and game species (as stated in Kazimirov et al. 2022). Despite the biological resiliency of wolves that often allows them to survive persecution, humans have proven capable of drastically reducing wolves from both rural and developed areas (Mech 1970). RK Wayne’s work deals with the discrepancy between biological considerations and the conservation of wolf ESUs in practice, in particular for North America (Hendricks et al. 2019). Our review indicates that similar inferences could be made for Asian ESUs of wolves. Finally, there are also learned traits, which may arise locally and characterize wolf ecotypes, and research could focus on such aspects of canid “culture” to also maintain.

### Conducting research on wolves using novel molecular tools that challenge conservation genetics paradigms

RK Wayne has demonstrated that when research on wolves is supported by novel molecular tools, its findings will and should challenge important conservation genetics paradigms. For instance, a paradigm of conservation is that small populations face high extinction risks due to the combined effects of random drift, fixation of deleterious mutations, and inbreeding depression (Frankham 2005). Nevertheless, we know a number of small populations that apparently are surviving well, and work by RK Wayne and colleagues emphasizes the power of genomics to evaluate the balancing consequences of some unexpected evolutionary factors in small canid populations (Mooney et al. 2023). A new theory is therefore emerging: that, in the long-term, small populations may more efficiently purge deleterious mutations, thus mitigating the consequences of inbreeding on fitness. Therefore, in some cases, long-term demographic patterns, also including ancient and recent bottlenecks, may be more consequential than inbreeding. This is just an example of research excellency, of creativity and of thinking outside the box, as RK Wayne was equally capable of research highlighting opposite trends, for example, using new genomic tools he detected signatures of extensive inbreeding in wolves, and claimed that the small Isle Royale population was on the threshold of extinction (Robinson et al. 2019). Additional research also supported by novel tools will likely challenge current paradigms regarding genetics and viability of small populations and hopefully arrive at new syntheses that also have practical applications for conservation.

### Formulating innovative views on hybridization in *Canis*, including potential fitness benefits of wild wolf × domestic dog introgression

Innovative views are required on the creative role of interspecific hybridization in *Canis* species, including the rather heterodox view of potential fitness benefits deriving from wolf-domestic dog admixture and adaptive introgression. The evolutionary outcomes and conservation implications of hybridization are often controversial. Therefore, RK Wayne claimed that, in the Anthropocene context, it is appropriate to distinguish “natural” hybridization events from the “anthropogenic” ones, that is, those directly determined or indirectly favored by human impacts on populations and ecosystems (Wayne and Shaffer 2016). While interspecific gene flow through hybridization might have had a creative role in evolution, crossbreeding of wild canids with domesticated dogs, which is widespread in various parts of the world, constitutes one of the main risks for the conservation of natural populations of *Canis* (Pilot et al. 2018, 2021). In general, anthropogenic hybridization is thought to have mainly negative consequences on the fitness of the hybridizing wild populations (Wayne and Shaffer 2016).

RK Wayne dedicated a sizeable part of his career to study hybridization in *Canis*, applying, in synchrony with the development of new technologies, different approaches from mtDNA typing, to microsatellites and single-nucleotide polymorphisms (SNPs) genotyping, to whole-genome analyses (i.e. Wayne and Jenks 1991; Chavez et al. 2022). His team also provided a rationale for the conservation of hybridizing populations, if threatened (Wayne and Shaffer 2016; vonHoldt et al. 2017). Hybridization and admixture may maintain genetic diversity, preserving the adaptive and evolutionary potential of populations, thus allowing some species to keep serving important roles in ecosystems. Admixture, either natural or artificial via translocations, could be the only way to prevent the extirpation of some inbred populations. In these cases, hybridizing populations could in theory be protected even by law (Allendorf et al. 2001). However, the legal frameworks regarding hybrids may be unclear and inadequate (Trouwbost 2014).

## Ecological specialization of ecotypes in northern North America

Research by RK Wayne’s group has been seminal in unraveling the geographic partitioning of genetic diversity among wolf populations. Early studies of mtDNA sequences or microsatellite loci identified genetic partitioning (Roy et al. 1994; Vilà` et al. 1999). Then, vonHoldt et al. (2011) used over 48K SNP loci in “wolf-like” species. Patterns in this genomic variation revealed distinct genetic clusters for North American, Asian, and European populations, also with a sharp distinction of Spanish and of Italian peninsular wolves. Overall, the study by vonHoldt et al. (2011) showed that, despite the species’ high mobility and long-range dispersal, wolves were structured in distinct units, which aligned with geographic distribution and local ecological conditions.

Unlike wolves in the southern part of the species’ distribution, Holarctic wolf populations have occupied vast regions of northern North America and the Old World, virtually unfragmented by human impacts or climatic events since the last glaciation. The pattern of “population continuity” exhibited by wolves may be atypical when compared with humans, a variety of vertebrate taxa, and vascular plants, a notion which is highlighted in Schweizer and Wayne (2020). In these regions, ecological specialization has resulted in genomic differentiation of wolf ecotypes (Leonard 2015; Hendricks et al. 2019). Theoretically, both barriers to dispersal and climatic and habitat (ecological) differences will determine differentiation of any species. However, in the absence of barriers, ecological factors may still cause genetic differentiation, as wolves primarily disperse within their natal habitat (Leonard 2015). Various works lead by RK Wayne (listed below) therefore arrived at a synthesis, that, in relation to climatic factors and specialization on their main prey, northern North American wolves formed High Arctic, Arctic, Boreal Forest, Forest, and Coastal ecotypes.

**Fig. 1.**
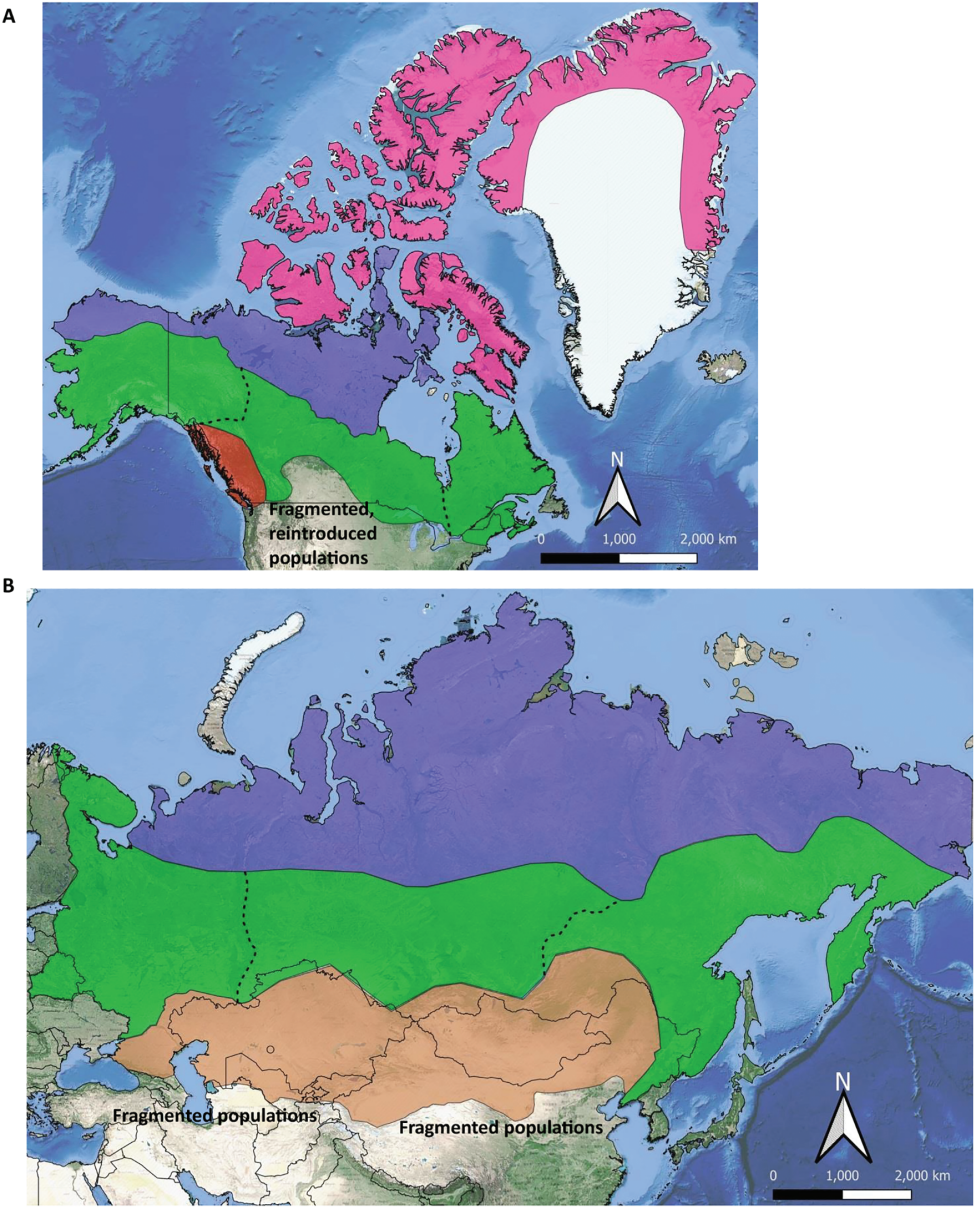
In North America A), and Asia B), Arctic wolves occur in the barren-ground tundra and in the northern taiga areas frequented by migratory caribou or reindeer, respectively (blue polygons). Forest wolves live in forested habitats (green polygons) and different ecotypes may be distinguishable (broken lines), depending on the forest type and prey base. In North America A), High Arctic wolves also occur in the tundra of the Arctic Archipelago and Greenland (pink polygon), relying on residential prey ranging in size from hares to muskoxen; and Coastal wolves (red polygon) rely mostly on just one deer species and a vast array of marine resources. Finally, in Asia B) a genetically distinguishable Steppe Wolf might also be present that relies on wild and domestic ungulates (brown polygon).

**Fig. 2.**
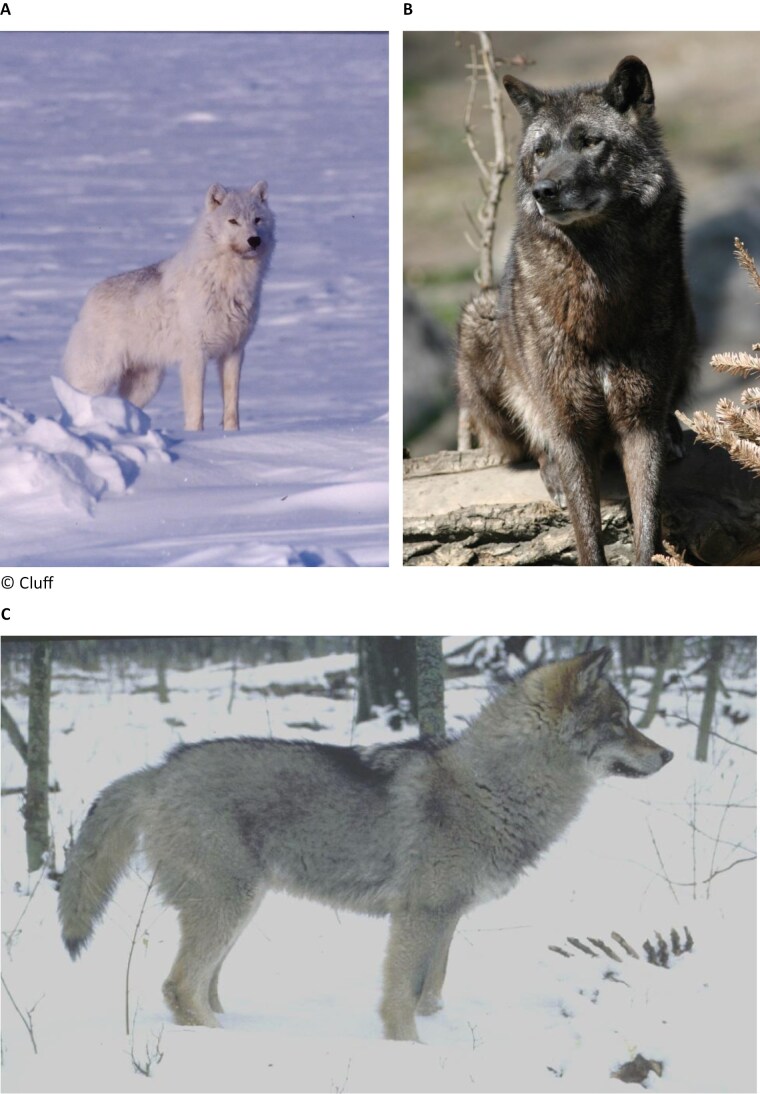
Typical coloration of wolves including A) a wolf belonging to the Arctic ecotype, which occurs in the continental tundra of North America, and in the sparsely treed northern fringes of the taiga, B) a wolf belonging to the Boreal Forest ecotype, which lives in forested habitats of North America dominated by coniferous tree species, and C) a young wolf belonging to the Forest ecotype, which lives in forested habitats of Europe and Asia with some local additional differentiation also possible, depending on the forest type and prey base.

Multiple studies identified patterns of genetic variation that were associated with ecological differences in wolves. Two works identified genomic evidence for ecotype differention in northern North American wolves (Schweizer et al. 2016a, 2016b), and other works correlated genetic to ecological variation of the species (see e.g. Carmichael et al. 2007; Musiani et al. 2007; Muñoz‐Fuentes et al. 2009; Fig. 1A). Such studies determined that High Arctic wolves occur in the barren-ground tundra of the Arctic Archipelago and likely Greenland, are mostly white or pale in coloration (they live in areas that are snow covered for long periods of the year), rely on residential prey ranging in size from hares (*Lepus arcticus*) to muskoxen (*Ovibos moschatus*) and occupy large home ranges year-round. High Arctic wolves are genetically distinct from Arctic wolves that occur in the continental tundra and the sparsely treed northern fringes of the taiga. Both High Arctic and Arctic wolves are similarly colored (Fig. 2A), however, Arctic Wolves specialize on barren-ground caribou (*Rangifer tarandus)* and follow the migrations of caribou herds (i.e. a behavior first documented by RK Wayne’s group; Musiani et al. 2007). Boreal Forest and Forest wolf ecotypes are also genetically distinguishable, inhabiting forests dominated by coniferous or deciduous tree species, respectively. Forest wolves are mostly gray or dark (Fig. 2B), and primarily rely on several species of residential Cervids but are nonmigratory. Finally, Coastal wolves are morphologically similar to forest wolves but rely on a vast array of marine resources, including marine mammals and seasonally spawning salmon, in addition to a single species of deer (typically Black-tailed deer, *Odocoileus hemionus columbianus*; Muñoz-Fuentes et al. 2009).

Ecological variation was likely accompanied or driven by genomic variation. Schweizer et al. (2016b) characterized a number of candidate genes under selection in wolves which were associated with the environment and climate of the regions occupied by each ecotype. Additionally, the genes that were under selection had functions that likely explained known differences in morphological, ecological, and behavioral traits of wolves (Schweizer et al. 2016a). Differences in sizes between wolf ecotypes were evident (O’Keefe et al. 2013), likely explained by Bergmann’s (1847) rule that body size increases with latitude (larger body sizes conferring a thermoregulation advantage in colder climates). Other differences could be correlated to both water precipitation and primary productivity (which vary between the ranges used by continental and more coastal ecotypes), and other differences to prey availability, which in turn may affect skull morphology.

The wolf ecotypes described so far likely formed during a period starting approximately 23 ka, when North America was colonized by wolves from Beringia. These time frames were more recent than previously thought, but are supported by whole mitochondrial genomic analyses (Koblmüller et al. 2016). Furthermore, full genome sequence analysis confirmed the recent common ancestry of all extant wolves (sampled in both North America and Eurasia; Fan et al. 2016).

## Old-World ecotypes: preliminary data indicate similar patterns

We analyzed the literature available on wolves for Asian regions of the Old World, which similar to the northern part of North America are sparsely populated by humans. We suggest that patterns of specialization and differentiation of wolf ecotypes that are similar to those described by RK Wayne’s group might also be present in Asia, although genetic evidence for these might be still incomplete (Poyarkov et al. 2022; Fig. 1B). Overall, Crandall et al.’s (2000; note that RK Wayne was an author of the study) integrative approach toward defining ESUs might also be valid for wolves in the Old World as well as northern North America, as ecotypes differ depending on genetic, morphological, ecological, and behavioral traits.

Nowak (2003) first indicated the likely organization and distribution of wolves in Asia, largely based upon morphological observations, particularly skull measurements. Asian authors often write of wolf “subspecies” and not about ecotypes, two concepts which were compared/contrasted starting with the first paper on ecotypes (Gregor 1944). However, Asian authors also emphasize wolf differentiation based upon ecological differences (similar to RK Wayne and coauthors for North America), and therefore we use the term ecotypes for such groups of wolves. Ersmark et al. (2016) and Vilà et al. (1999) evaluated wolf phylogeography globally based on the mitochondrial control region, using samples also from Eurasia. More recently, Poyarkov et al. (2022) provided an overview of current research on wolves in Russia and the former USSR, therefore including much of the species range in the Old World. Finally, Werhahn et al. (2022) reviewed all data available on Asian wolves, including genetic, morphological, ecological, and behavioral traits. Such studies are consistent with Kazimirov et al. (2022), which deals with the concepts of subspecies and ecotype, and summarizes “the most accepted system of the subspecies” in Old-World Holarctic wolves. This system includes the tundra wolf *C. lupus albus*, the forest wolf *C. l. lupus*, the Caucasian wolf *C. l. cubanensis*, the steppe wolf *C. l. campestris*, the Mongolian/Tibetan wolf *C. l. chanco*, and the desert wolf *C. l. desertorum*. However, Talala (2020), based both on the ecological literature (e.g. Suvorov and Kirienko, 2008) and on genetic differentiation data, arrive at a synthesis of ecological specialization of wolf ecotypes that is similar to RK Wayne’s findings for northern North American wolves.

Our overview of the literature indicates that Asian Arctic wolves occurring in the barren-ground tundra and in the northern taiga (often referred to as Tundra wolves or *C. l. albus*) are mostly white or pale in coloration, like the Arctic wolves of North America studied by RK Wayne. The southern range of tundra wolves reaches the southern boundary of the winter distribution of the tundra populations of wild reindeer, *R. tarandus*. This association between tundra wolves and their main prey and its range is identical to what found by Musiani et al. (2009) for North American wolves and caribou. It should be noted here that the northern part of Eurasia does not include an archipelago, similar to the one that promoted wolf ecotype differentiation in North America. Forest wolves (*C. l. lupus*), which inhabit forested habitats are also mostly gray or dark and primarily rely on several species of residential Cervids. It was also suggested that, similar to North America, different ecotypes of Forest wolves may be distinguishable, depending on both forest and prey type. Thus, specialization of ecotypes could depend upon main prey, including moose (*Alces alces*) in western forests, forest reindeer in eastern forests (i.e. potential *C. l.* var. *orientalis* ecotype), Siberian elk (*Cervus elaphus sibiricus*) in the Sayan Mountains taiga (i.e. potential *C. l. altaicus* ecotype; Fig. 1B).

The population genetics of a Steppe Wolf ecotype (*C. l. campestris;*  Kazimirov et al. 2022), has also been studied, which possesses a lighter and reddish coloration. Interestingly, the Latin name used for this Steppe Wolf was identical to the wild female wolf from Mongolia for which the complete mitochondrial genome was analyzed by Zhang et al. (2015). Neither study examined the differentiation of Steppe wolves from other ecotypes, one used neutral genetic markers only and found significant gene flow from neighboring regions (Kazimirov et al. 2022), while the other did not compare results to other locations. However, Stronen et al. (2015) found differentiation in functional genes between wolves from the Ukrainian Steppe (i.e. an area of steppe with fragmented wolf populations in our Fig. 1B) and wolves from other European countries. Overall, although in-depth genetic studies on wolves in Central Asia, Pakistan, Mongolia, eastern Russia, and China are still required Werhahn et al. (2022), the possibility of a Steppe Wolf frequenting central Asia (and reaching to the Ukraine) and relying on both wild and domestic ungulates should not be neglected (Fig. 1B).

As in North America, morphological differences are noticeable between Asian wolf ecotypes (see Kazimirov et al. 2022) also with body size increasing with latitude. However, studies correlating genomics to gene expression and function, similar to those conducted in North America by RK Wayne, are still lacking for Asian wolves, which impedes a comprehensive characterization of ecotypes or ESUs. Finally, data gathered across Europe could support the existence of genetic differentiation of wolf ecotypes there too (Stronen et al. 2013). However, at present, any identification of patterns of adaptation to local environments may be confounded by human-related factors such as landscape fragmentation and historical killing of wolves in some areas (Stronen et al. 2013).

## Challenging wolf conservation genetics paradigms in south-eastern Asia

RK Wayne’s work shed light on the evolutionary biology of Asian wolves (Zhang et al. 2014; Fan et al. 2016; vonHoldt et al. 2017). Despite this, the phylogeny, systematics, and taxonomy of Asian wolves remain unclear, largely due to insufficient geographic sampling, genetic consequences of habitat fragmentation, human-caused population declines, and local extirpations. As an example, Shrotryia et al. (2012) and Werhahn et al. (2022) reconstructed the historical debate on the confusing wolf systematics in the Indo-Himalayan-Tibetan region. Early taxonomists described two morphologically distinct wolf taxa, which were widely distributed throughout the region: 1) the lowland Indian wolf *Canis pallipes* (or *C. lupus pallipes)* occurring in the Indian subcontinent and closely related to other wolf populations with uncertain taxonomy also occurring in the Middle East, Arabia and eastern North Africa; and 2) *Canis chanco* (or *C. l. chanco*), occurring across a long geographical belt running from Manchuria to Pakistan (i.e. an area bordering or overlapping with that of the previously mentioned Steppe Wolf). The highland dwelling, woolly *chanco* wolf has also been described as a distinct species, *C. laniger*. However, phylogenetic studies were not conclusive, and suggested biased sampling, admixture, and differential gene introgression among these wolves (Sharma et al. 2004; Aggarwal et al. 2007; Chetri et al. 2016; Werhahn et al. 2018, 2020; Joshi et al. 2020). Finally, genomic analyses by Hennelly et al. (2021) demonstrated that *chanco* and *pallipes* wolves were distinct and basal to all Eurasian (Holarctic) wolves, and evidenced admixture between the two taxa (Werhahn et al. 2020).

RK Wayne’s research supports the genetic and functional uniqueness of *chanco* wolves. It showed that highland woolly (*chanco*) wolves that inhabit altitudes higher than 4,000 m in the Himalayas, Tibet, and Mongolia were enriched for hypoxia-related genes and thus were likely adapted to hypoxia in these low oxygen tension ecosystems (Zhang et al. 2014). Using genome sequencing, Wang et al. (2020, 2022) then highlighted the admixed ancestry of *chanco* wolves, which could explain the inconsistent phylogenies obtained previously, and suggested that an unidentified and probably extinct “ghost” wolf population transmitted much of their genomes to the extant *chanco* wolves. The EPAS1 haplotype adaptive to hypoxia and prevalent in *chanco* wolves might have been derived from the ghost lineage.

The genetic and ecological diversity of wolf populations in lowland China and in East Asia remains largely unknown, although wolves may still be wide-ranging in the region, even after the sharp growth in human density and infrastructure in recent years. Wang et al. (2019) using genomic data from a few museum samples showed that East Asian wolves may split in three groups distributed in Southern China, Northern Asia, and Tibet, respectively. However, such groups were considerably admixed. Finally, phylogenetic studies have clarified the origin of the two distinct wolf lineages that went extinct in Japan c. 120 to 140 years ago: the Japanese or Honshu wolves (*C. l. hodophilax*) from the Kyushu, Shikoku, and Honshu islands, and the Ezo or Hokkaido wolves (*C. l. hattai*) from the island of Hokkaido (Matsumura et al. 2014). The Hokkaido wolves, genetically and morphologically related to Canadian wolves, could have colonized Hokkaido from Siberia via the Sakhalin Island, which were bridged by land before the LGM (c. 10,000 YBP).

## Challenging wolf conservation genetics paradigms in European and Middle Eastern countries

In Europe, wolves have been persecuted for centuries, and as a result populations were reduced or eradicated, except in some Eastern European countries. By the mid-1900s, wolves survived only in a very small population totally about 100 individuals in the central-southern Italian Apennine mountains, in a few fragmented populations in the Iberian Peninsula, and in relatively more abundant populations in Greece and the Western Balkans. Wolves were eradicated from France, from the Alps, from Germany, and the northern EU countries between the end of the 1800s and the first two decades of the 1900s. Consequently, the phylogeographic patterns of European wolves have been deeply confounded by human impacts. Furthermore, loss of genetic diversity and inbreeding likely contributed to local extinctions. Finally, in southern European countries, the concomitant expansion of feral domestic dogs resulted in hybridization and introgression of DNA from dogs (see references in Randi 2011). However, the socio-economic and human demographic changes which transformed the European countries between the 20th and 21st century, boosted deep ecological transformative processes as well. Consequently, wolves rapidly recolonized even their northernmost historical ranges from their refugia. The expansion of Italian wolves exemplifies this: in less than 20 years (max. 4 to 5 wolf generations) wolves coming from the Apennine mountains colonized the western Alps and southwestern France by 1992, then crossed to Switzerland in 1994–1995, and further expanded westward reaching the Pyrenees and Catalonia (south-eastern Spain) by 2000. Such range expansion was spearheaded by long-range dispersal (Valiere et al. 2003; Ciucci et al. 2009). Early colonizers often went unnoticed, and in some cases, there was suspicion that wolves were illegally translocated far from their natural source populations or even worse: that captive wolves of unknown origin had been released. In such situations, diagnostic molecular tools were often useful in providing an improved narrative (Fabbri et al. 2007).

A mitogenomic study (Doan et al. 2023), which was coauthored by M. Pilot, RK Wayne’s collaborator on several European and Middle Eastern works, expanded on previous findings (Pilot et al. 2010) showing that Eurasian wolf populations bear two haplogroups: W1, frequent in modern wolves; and W2, frequent in ancient wolves. Haplogroup W1 has been found in all Holarctic wolves (i.e. also in North America), probably originated in Northern Siberia, and spread westward into Europe about *c*. 23,500 years ago (Loog et al. 2020). Therefore, the ancestral haplogroup W2, which had originated in Europe previously (i.e. *c*. 35,000 years ago), has been largely replaced by W1’s expansion. However, interestingly, the current expansion wave of Italian wolves, that harbor W2 haplotypes, is restoring at least in part the genomic diversity that had been lost in ancient and historical times.

Genomic research (Pilot et al. 2014a; Silva et al. 2020) revealed that wolf populations were likely isolated during the last c. 10,000 years in southern European refugia. These were likely the ancestors of most wolf populations now living in the EU. Dufresnes et al. (2018), Hindrikson et al. (2017), and Stronen et al. (2013) concur that extant wolves in the EU can be divided in six major clusters: Italian, Dinaric-Balkanic, Carpathian, Ukrainian, north-central European, and Iberian. On the other hand, the population in the Baltic countries is widespread and likely connected with wolves in western continental Russia, Finland, Poland, Ukraine, and Belarus (Baltrūnaitė et al. 2013).

At present, the wolf is protected by law in many European countries. Habitat is suitable, and prey availability is sustaining wolf expansion, facilitating the reconnection of population fragments. In particular, wolves are still expanding in central and eastern Europe, and their population genetic structure is dynamic (Gula et al. 2020; Jaraush et al. 2021; Szewczyk et al. 2021). The colonization of north-western Europe started even more recently (from a decade ago; Gravendeel et al. 2013; Andersen et al. 2015). Finally, admixture from historically isolated populations is regenerating genetic variability, which in turn is likely enhancing fitness (genetic rescue effect; Fabbri et al. 2014). Thus, in the last 10 years, wolf range has increased by 25%, with total number of wolves in Europe surpassing twenty thousand in 2022 (Cimatti et al. 2021).

Both the distribution and diversity of wolves in the Near and Middle Eastern countries are poorly understood. However, research is ongoing, and genetic evidence gathered thus far indicates that the Caucasian countries may be transitional between East European and Middle Eastern wolf populations (Pilot et al. 2014b). Data available on wolves from the Arabian Peninsula and Iran do not allow conclusions on population diversity or taxonomic status. Furthermore, similar to European populations, these populations may also be threatened by hybridization with domestic dogs (Khosravi et al. 2013; Kopaliani et al. 2014; Pilot et al. 2014b).

## The consequences of small population size: new lessons from Scandinavia

Early research by RK Wayne and group was seminal in using molecular analyses to highlight conservation concerns for wolves due to both hybridization and habitat fragmentation (Wayne et al. 1992; Roy et al. 1994). In particular, the consequences of inbreeding, including inbreeding depression and fixation of deleterious mutations, have been a concern throughout his career (Robinson et al. 2019; Kryazis et al. 2020), and for such matters the Scandinavian wolves are exemplary. The Scandinavian wolf population was extirpated by 1966, and then reestablished by a single pair immigrating from the bordering Finnish-Russian population (Vila et al. 2003). These founders were related likely due to their origin from a historically declining source population (Viluma et al. 2022) and despite small amounts of immigration, the Scandinavian population, now numbering c. 480, is characterized by the effects of small effective population size (Liberg et al. 2005; Bensch et al. 2006). Following the arrival of two new immigrants in 2008, the population showed some signals of genetic rescue, and the most heterozygous resident wolves were the most efficient breeders (Bensch et al. 2006). However, Scandinavian wolves still have long runs of homozygosity (Kardos et al. 2018), and increasing values of inbreeding (Åkesson et al. 2022; Viluma et al. 2022). As a result, morphological anomalies potentially affecting fitness (Räikkönen et al. 2013), and decreases in fertility, birth rate, litter size, and yearling survival have all been observed (Åkesson et al. 2016), implying that more immigrants are urgently needed.

In wolves, inbreeding among close relatives may be rare (Mech, 1970) and selective mating might favor heterozygosity (Bensch et al. 2006). However, the consequences of inbreeding in small, isolated populations of wolves are pervasive, including reduced growth rate, with effects on viability, and ultimately persistence (see RK Wayne’s analysis in Robinson et al. 2019). If conservation is a societal concern, then wolf populations should be managed to allow gene flow from genetically viable source populations. Furthermore, both source and receiving populations should be monitored, and causal correlations between inbreeding and fitness should be estimated across generations. As RK Wayne’s studies indicated, if the source populations are also inbred or historically declining (Viluma et al. 2022), then any positive consequences or “genetic rescue” due to gene flow could be invalidated by the diffusion of lethal or deleterious mutations, with receiving populations becoming sinks (Kryazis et al. 2020). Overall, the Scandinavian findings emphasize the need for transboundary wolf conservation plans promoting connection of functioning biological populations, across jurisdictional borders (Linnell et al. 2008).

## Evolution and conservation of wolf-dog hybrids

Hybridization, the breeding of individuals belonging to taxonomically distinct species, is a word with many meanings and includes a multifaceted set of complex events whose dynamics at the genomic, demographic, and ecological level are still poorly understood. Hybridization may occur naturally, or may be human caused. Almost all living species of *Canis* experienced repeated episodes of natural hybridization and adaptive introgression during their evolution (Gopalakrishnan et al. 2018). Wolves are the ancestors of domesticated dogs, and wolf-dog hybrids are fully viable and fertile (Mech 1970). Recent hybrids and backcrosses up to the fourth generations are reliably identifiable by genotyping small panels of ancestrally informative microsatellites or SNPs. Conversely, the identification of older introgressions is more complex and requires the use of genomic analyses. Finally, the identification of molecular markers associated with morphological variants of documented domestic origin, as well as of diagnostic mtDNA and Y chromosome haplotypes, facilitates the identification of individuals of admixed ancestry (Galaverni et al. 2017).

In Europe, hybridization between wolves and dogs is widely reported, but with highly variable prevalence between regions, probably in relation to differences in management of stray dogs and varying densities of dog populations (Pilot et al. 2018). Despite the conservation concerns regarding hybridization, the prevalence of hybrids has been estimated only opportunistically, through insufficient sampling schemes and by heterogeneous panels of molecular markers (Stronen et al. 2022a). The frequency of first-generation hybrids and backcrosses has been crudely estimated to range between 5% and 10% in various European countries (Godinho et al. 2011; Randi et al. 2014). However, in some localities where the presence of hybrid packs was of particular concern, higher frequencies have been also estimated (20% to 50%; Salvatori et al. 2019). By comparison, evidence of wolf-dog hybridization in Eastern and Asian countries is limited and data are not enough to evaluate distribution of hybrids (Werhahn et al. 2020 for the Himalayas; Khosravi et al. 2013 for Iran; Kopaliani et al. 2014 for Armenia).

A conspicuous phenotypic trait introgressed in wolves via past hybridization with dogs is the black coloration. Such introgression and phenotype were first detected in a number of North American wolf populations by RK Wayne’s group (Anderson et al. 2009). They were then sporadically identified in some Eurasian populations (i.e. rare occurrence of black individuals), but, as far as we know, they were common only in some areas of the central-northern Italian Apennines (Randi 2011). Based on a seminal study by Candille et al. (2007), RK Wayne and coworkers were the first to identify a causal relationship between a 3 bp deletion at the β-defensin *CBD103* gene (the dominant melanistic *K*^*yB*^ allele at the *K locus*) and black coloration (Anderson et al. 2009). This structural variant was also unequivocally associated with black coloration in European wolves (Galaverni et al. 2017). The molecular evidence for a selective sweep was at first explained ecologically: that a black coloration conferred adaptive advantages to wolves living and preying in dark forest environments via camouflage, compared with others living in more open environments. However, the hypothesis of camouflage has not stood up to critique, resulting from in-depth research on wolves reintroduced in Yellowstone National Park (Hedrick et al. 2016). Alternative hypotheses for the increased fitness of black wolves include assortative mating between black and gray wolves, or black wolves’ resistance to parasitic and infectious diseases through pleiotropic mechanisms (Cubaynes et al. 2022).

Thus, hybridization studies have clarified fundamental aspects of evolution and adaptation, while also contributing to conservation in practice. Wolves have hybridized with dogs since at least 14,000 years ago (Anderson et al. 2009; Doan et al. 2023), and the resulting introgression has produced phenotypes that have survived in wild wolf populations since then. Current and future conservation practices, also including, for example, the iconic reintroduction of wolves to Yellowstone, may account for wolves that are black and dog-introgressed too—that is, being considered entirely natural. Overall, it is not clear whether the assumption of conservation biology, that anthropogenic hybridization is a risk factor for natural populations, is always supported by empirical evidence and is generalizable (Allendorf et al. 2001). Therefore, formulation of conservation solutions requires scientific research and in-depth analysis of case studies involving hybridization, debating of any findings by academics and all conservation stakeholders, and the development of monitoring and management procedures applicable in the real world of conservation, a world that is perhaps more inclusive toward hybrids.

## Data Availability

No data are associated with this article.
